# Long-term outcomes of 0.1% tacrolimus eye drops in eyes with severe allergic conjunctival diseases

**DOI:** 10.1186/s13223-021-00513-w

**Published:** 2021-02-01

**Authors:** Hiroyuki Yazu, Kazumi Fukagawa, Eisuke Shimizu, Yasunori Sato, Hiroshi Fujishima

**Affiliations:** 1 Department of Ophthalmology, Tsurumi University School of Dental Medicine, 2-1-3 Tsurumi Tsurumi-ku, Yokohama-shi, Kanagawa, 230-8501 Japan; 2 Department of Ophthalmology, Keio University School of Medicine, Tokyo, Japan; 3 Ryogoku Eye Clinic, Tokyo, Japan; 4 Department of Preventive Medicine and Public Health, Biostatistics At Clinical and Translational Research Center, Keio University School of Medicine, Tokyo, Japan

**Keywords:** Tacrolimus, Atopic keratoconjunctivitis, Vernal keratoconjunctivitis, Objective sign, Steroid, Intraocular pressure

## Abstract

**Background:**

Because atopic dermatitis does not heal completely, associated severe atopic keratoconjunctivitis (AKC) and vernal keratoconjunctivitis (VKC) often require long-term treatment. This study aims to evaluate the long-term outcomes of using 0.1% tacrolimus eye drops to treat these severe allergic conjunctival diseases.

**Methods:**

Two-hundred-and-seventy eyes of 135 patients diagnosed with AKC or VKC from April 2004 to April 2014 were screened retrospectively. Patient demographics and objective signs were extracted from the electronic medical records. The severity of 10 objective signs, related to the palpebral and bulbar conjunctiva, limbus, and cornea, and intraocular pressure (IOP) were observed at baseline, at 2 weeks, 1, 2, 3, 6, and 12 months after starting treatment, and every 1 year thereafter (average use period: 8.4 ± 2.9 years). Safety was evaluated based on the incidence and severity of adverse events.

**Results:**

12 patients (AKC; 7 cases, VKC; 5 cases) who were treated with 0.1% tacrolimus eye drops were enrolled in this study. The total score of clinical signs significantly decreased after 2 weeks and remained effective thereafter. Tacrolimus eye drops elicited a statistically significant difference in the mean total clinical scores and IOP over the course of treatment (P < 0.001). Elevated IOP was observed in 2 cases and corneal infection in 1 case; these effects were completely controlled with medication.

**Conclusions:**

Topical tacrolimus may provide effective and long-term improvement in clinical signs of severe AKC and VKC cases that refractory to standard conventional treatment.

*Trial registration*: University Hospital Medical Information Network (UMIN) 000034460.

## Background

Allergic conjunctival diseases (ACDs) are broadly divided into five types according to the presence of proliferative changes, atopic dermatitis (AD), and the presence/absence of mechanical irritation (i.e., seasonal allergic conjunctivitis (SAC), perennial allergic conjunctivitis (PAC), atopic keratoconjunctivitis (AKC), vernal keratoconjunctivitis (VKC) and giant papillary conjunctivitis (GPC)). AKC and VKC are categorized as severe types of ACDs, because they can cause keratopathies [1], such as shield ulcer or corneal plaque, leading to visual morbidity in some cases [2, 3]. Hence, appropriate treatment methods have been required to avoid declines in quality of life and vision. Chronic allergic conjunctivitis can contribute to development of AKC and VKC [4], and are characterized by the mucosal infiltration of eosinophils, neutrophils, basophils, mast cells, and T lymphocytes [5, 6]. Moreover, subepithelial fibrosis of the conjunctiva, shortening of the fornix, and symblepharon may occur in the most severe cases [7]. In the treatment of AKC and VKC, topical antihistamines, mast cell stabilizers, non‐steroidal anti‐inflammatory drugs (NSAIDs), cyclosporin, and steroids are usually used. However, we have encountered patients who are refractory to these medications. Although systemic steroids may be required to relieve patient symptoms, they can cause adverse effects, such as cataract, infection, steroid-induced elevation of intraocular pressure (IOP), glaucoma, eyelid skin atrophy, and depigmentation with barrier impairments [8–10]. To overcome these issues, alternative therapeutic medications for AKC and VKC are required. Recently, tacrolimus has been used as a 0.1% ophthalmic suspension for treatment of several ocular diseases [11–15]. In these previous reports, however, the observation period was short (less than 1 year follow up study) and/or mild cases without corneal involvements were included. We consequently investigated the therapeutic effects of topical tacrolimus for severe AKC throughout a 1-year period [16]. Based on our clinical experience, because atopic dermatitis cannot be completely cured, the associated severe AKC and VKC often required even more prolonged treatment. As a next step, this study examined whether the efficacy and safety of tacrolimus could be maintained by investigating the long-term use of 0.1% tacrolimus eye drops in severe ACDs.

## Methods

### Atopic keratoconjunctivitis and vernal keratoconjunctivitis

According to definitions in Japanese guidelines [17, 18], most AKC patients experience no proliferative changes, but most cases of VKC demonstrate proliferative changes, such as cobblestone papillae of the palpebral conjunctiva, accompanied by AD. Diagnoses of these conditions are thus based on these guidelines [17, 18], but the borderline for distinguishing between AKC and VKC is vague in actual clinical practice. Thus, we diagnosed AKC as chronic keratoconjunctivitis associated with AD on the patient’s face, according to the following criteria: (1) it was always associated with other AD; (2) it occurred at any time during the associated atopic disease, independent of its severity; and (3) evidence existed for corneal involvement during the disease course [19], as in our previous study [16]. The diagnosis of AD was based on previously reported criteria [20]. Severe AKC and VKC were defined as conditions with corneal involvements.

### Study design

**Fig. 1:**
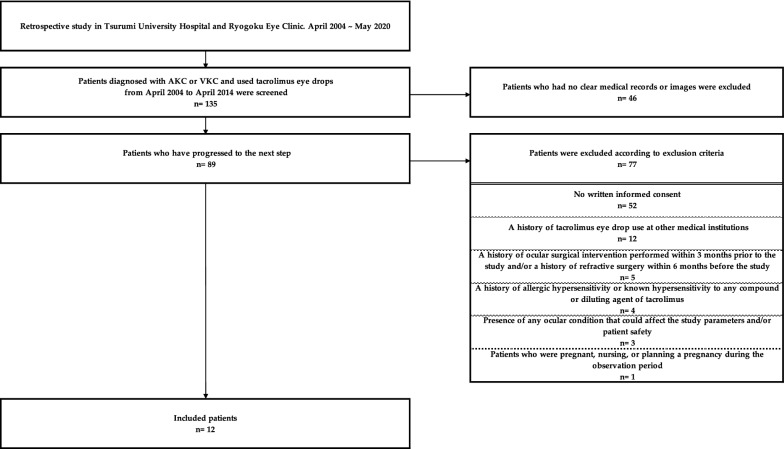
Study flowchart

This retrospective study was performed as summarized in Fig. 1. We screened 270 eyes of 135 patients who were diagnosed with AKC or VKC and treated with tacrolimus eye drops at the Department of Ophthalmology, Tsurumi University School of Dental Medicine, and Ryogoku Eye Clinic between April 2004 and April 2014. The inclusion criteria were (1) Japanese males and females, (2) cases with clear medical records or images. Among these cases, 89 patients (178 eyes) who were using tacrolimus eye drops for AKC or VKC were screened. Patients who had any one or more of the following criteria were excluded: (1) No written informed consent. (2) A history of tacrolimus eye drop use at other medical institutions. (3) A history of allergic hypersensitivity or known hypersensitivity to any compound or diluting agent of tacrolimus. (4) Presence of any ocular condition that could affect the study parameters and/or patient safety (e.g., glaucoma requiring medication or laser treatment, clinically significant blepharitis, uveitis, or pterygium). (5) A history of ocular surgical intervention performed within 3 months prior to the study and/or a history of refractive surgery within 6 months before the study. (6) Patients who were pregnant, nursing, or planning a pregnancy during the observation period. In total, 24 eyes in 12 patients were eventually studied.

This study was performed in accordance with the tenets of the Declaration of Helsinki and ethics approval was obtained from Tsurumi University Dental Hospital (Kanagawa Prefecture, Japan; IRB No.1312). The clinical trial registration ID number is University Hospital Medical Information Network (UMIN) 000034460. Patient data were anonymized before access or analysis.

### Clinical signs and criteria

The severity of objective signs and IOP were observed at baseline (before treatment) and after 2 weeks, 1, 2, 3, 6, and 12 months, and every 1 year thereafter (average tacrolimus use period: 8.4 ± 2.9 years). Ten objective signs were assessed using 4 grades (0 = normal; 1 +  = mild; 2 +  = moderate; 3 +  = severe), as in previous reports [16–18]: palpebral conjunctiva (hyperemia, swelling, follicles, papillae, and giant papillae), bulbar conjunctiva (hyperemia and chemosis), limbus (Trantas dot and swelling), and corneal epithelial disorder. These criteria are summarized in Additional file 1: Table S1. The efficacy endpoint was assessed based on the extent of change in the total score of objective signs at the end of treatment. IOP was measured with Goldmann applanation tonometry (HAAG-STREIT, BQ900.4.4, Köniz, Switzerland). Safety was assessed based on the incidence and the severity of adverse events (especially ocular infections and elevated IOP).

### Treatments

We defined baseline as the point of initiation of topical tacrolimus. In the current study, all eyes were treated with 0.1% ophthalmic suspension (TALYMUS®, Senju Pharmaceutical Co., Ltd., Osaka, Japan) alone or in combination with other topical antihistamines, mast cell stabilizers, and steroids after baseline, that is, the use of topical NSAIDs and topical cyclosporine was discontinued. These were administered 1 drop bilaterally 2–4 times daily at the discretion of the attending physician (K.F., H.F.; coauthors of this research and allergists). No patient was treated with subconjunctival triamcinolone acetonide injection, oral NSAIDs, immunomodulatory therapy (IMT), biologic response modifiers (BRMs), or allergen immunotherapy before and during the observation period.

### Statistical analysis

The data were analyzed using Prism version 8.00 (GraphPad Software Inc, San Diego, CA, USA) for MacOS (Apple Inc, Cupertino, CA, USA) and SAS version 9.4 (SAS institute, Cary, NC, USA). The scores of clinical objective signs and IOP were analyzed using a mixed-effects model and graphed using Excel for Mac (ver. 16.40; Microsoft Corporation, Redmond, WA, USA). To evaluate the time course difference in the total scores and the IOP between AKC group and VKC group, one-way analysis of variance with Dunn’s multiple comparison test was performed. Data are presented as adjusted means ± 95% confidence intervals (CI), ± standard deviation (SD), or ranges. P values less than 0.05 were considered statistically significant.

## Results

### Characteristics of patients with severe ACDs

Demographic information of all patients in this study is shown in Table 1. All patients had bilateral keratoconjunctivitis and AD. At the beginning of the observation period, 7 patients (58.3%) were diagnosed with AKC and 5 patients (41.7%) with VKC. Asthma and allergic rhinitis were present in 16.7% and 5.0% of patients, respectively. Among all patients, 83.3% had previously used topical antiallergic eye drops, 95.8% used topical steroids, 16.7% used topical NSAIDs, 41.7% used topical cyclosporine, 8.3% used tacrolimus lid ointment, and 8.3% used systemic steroids.

**Table 1:** Demographics of patients before initiation of topical tacrolimus

Cases, Eyes	12, 24
Sex, n (%)	
Male/Female	10 (83.3)/2 (16.7)
Age (y) ± SD	15.7 ± 8.5
Duration of disease (y) ± SD	4.3 ± 1.8
Type of allergic conjunctival diseases, n (%)	
Atopic Keratoconjunctivitis (AKC)	7 (58.3)
Male / Female	6 (86) / 1 (14)
Age (y) ± SD	17.9 ± 10.4
Period of onset (y) ± SD	9.7 ± 3.0
Vernal Keratoconjunctivitis (VKC)	5 (41.7)
Male / Female	4 (80) / 1 (20)
Age (y) ± SD	14.0 ± 5.9
Period of onset (y) ± SD	6.4 ± 0.7
Other allergic complications, n (%)	
Asthma	2 (16.7)
Rhinitis	6 (5.0)
Atopic dermatitis (AD)	12 (100)
Pretreatment, eyes (%)	
Topical antihistamine and/or mast cell stabilizer	20 (83.3)
0.1% fluorometholone (FLM)	20 (83.3)
0.1% betamethasone (BEM)	4 (16.7)
Topical NSAIDs	4 (16.7)
Topical cyclosporine	10 (41.7)
Tacrolimus ointment	2 (8.3)
Oral steroid	2 (8.3)

*SD* standard deviation, *NSAIDs* non‐steroidal anti‐inflammatory drugs

### Changes in the scores of objective clinical signs

**Fig. 2:**
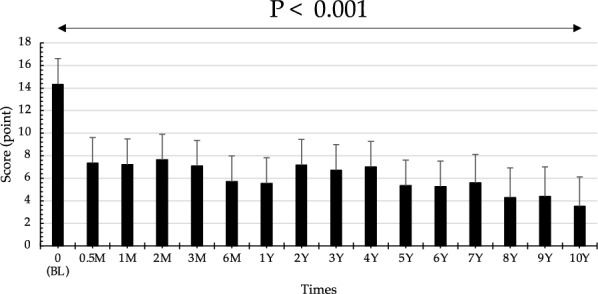
Changes in the scores of objective clinical signs after initiation of tacrolimus eye drop treatment. The total score of the 10 clinical signs (range 0–30) significantly decreased from baseline to 2 weeks after initiation of tacrolimus eye drop treatment. Tacrolimus eye drops elicited a statistically significant difference in the mean total clinical scores over the course of treatment. The adjusted mean score was 14.3 (95% CI, 12.1–16.6) at baseline and decreased to 3.49 (95% CI, 0.88–6.11) at 10 years after initiation

**Fig. 3:**
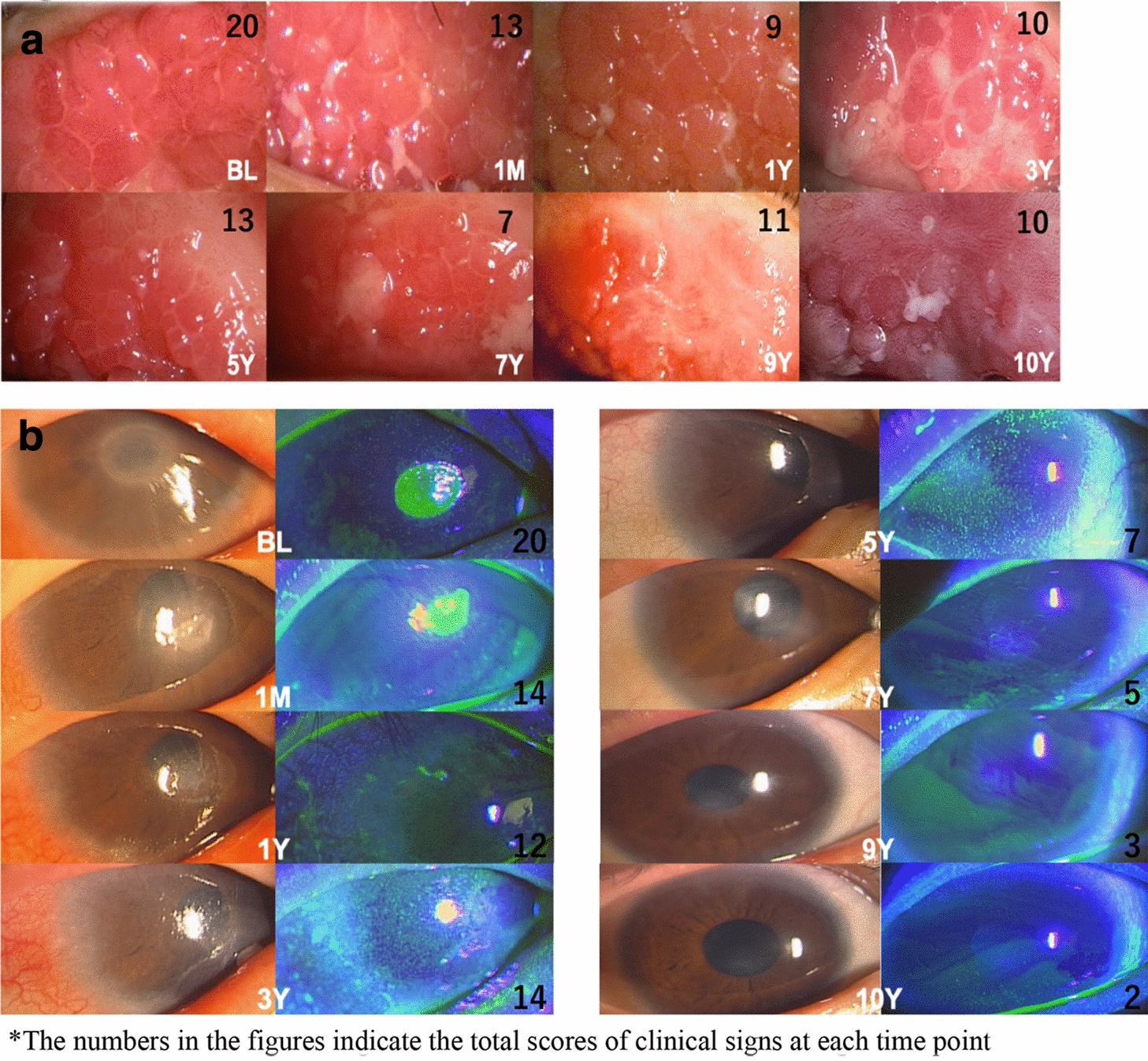
**a** Representative photographs of changes in the palpebral conjunctiva. An 11-year-old male patient was diagnosed with severe AKC, lasting 14 months, with giant papillae that almost entirely occupied the palpebral conjunctiva, with diameters exceeding 0.6 mm. He was treated with topical antihistamine, 0.1% betamethasone, and 0.1% cyclosporine in both eyes at his first visit to our outpatient clinic. A combination of topical 0.1% betamethasone and 0.1% tacrolimus eye drops was started to control the severe AKC. After 1 month, inflammation and proliferative changes began to decrease. Topical steroid use was discontinued after 6 months, but he continued tacrolimus treatment twice a day. One year after initiating treatment, the clinical signs of palpebral conjunctiva had gradually improved, and the elevated papillae became flat. However, because the patient’s compliance deteriorated, reactivation of AKC was observed at 5 years after initiation of tacrolimus eye drop treatment; thus, we increased tacrolimus treatment to 4 times a day. Finally, disease activity decreased, with some giant papillae and fibroproliferative changes noticeable after 10 years. **b **Representative photographs of changes in the bulbar conjunctiva, limbus, and cornea. A 12-year-old male patient was diagnosed with severe AKC, lasting 5 years, due to shield ulcer and bulbar conjunctival hyperemia with dilation of many vessels, thinner diffuse chemosis, Trantas dots, and swelling occupying two-thirds of the limbal circumference. He was treated with topical antihistamine, 0.1% fluorometholone, and 0.1% cyclosporine in both eyes at his first outpatient visit. A combination of topical antihistamine, 0.1% fluorometholone, and 0.1% tacrolimus eye drops was started to control the severe AKC. At 1 year after initiating treatment, the shield ulcer was completely resolved by using combination therapy. Topical steroid use was discontinued after 4 years, but he continued tacrolimus treatment twice a day. The bulbar conjunctival hyperemia disappeared after 5 years of treatment. Diffuse epithelial keratitis and corneal opacity also gradually improved and had almost disappeared by 10 years of treatment

The total score of the 10 clinical signs (range 0–30) significantly decreased from baseline (the adjusted mean score was 14.3; 95% CI, 12.1–16.6) to 2 weeks (7.33; 95% CI, 5.06–9.61) after initiation of tacrolimus eye drop treatment (Fig. 2, P < 0.001). Tacrolimus eye drops elicited a statistically significant difference in the mean total clinical scores over the time course of treatment (Fig. 2, P < 0.001). A comparison of the total scores of clinical signs is shown in Table 2. Representative photographs of changes in the palpebral conjunctiva are shown in Fig. 3a, and the bulbar conjunctiva, limbus, and cornea are shown in Fig. 3b (The numbers in the figures indicate the total scores of clinical signs at each time point).

**Table 2:** Comparison between time points in the total scores of clinical signs

Time	Number of patient (case)	Symptom Score (point)	SD	95% CI		Change from baseline (point)	SD	95% CI		P value*
0 M (BL)	12	14.3	1.09	12.1	16.6					
0.5 M	12	7.33	1.09	5.06	9.61	7.00	0.80	5.43	8.57	< .001
1 M	12	7.21	1.09	4.93	9.48	7.13	0.80	5.56	8.69	< .001
2 M	12	7.63	1.09	5.35	9.90	6.71	0.80	5.14	8.27	< .001
3 M	12	7.08	1.09	4.81	9.36	7.25	0.80	5.68	8.82	< .001
6 M	12	5.71	1.09	3.43	7.98	8.63	0.80	7.06	10.2	< .001
1Y	12	5.54	1.09	3.27	7.82	8.79	0.80	7.23	10.4	< .001
2Y	12	7.17	1.09	4.89	9.44	7.17	0.80	5.60	8.73	< .001
3Y	12	6.71	1.09	4.43	8.98	7.63	0.80	6.06	9.19	< .001
4Y	12	7.00	1.09	4.73	9.27	7.33	0.80	5.77	8.90	< .001
5Y	12	5.33	1.09	3.06	7.61	9.00	0.80	7.43	10.6	< .001
6Y	12	5.25	1.09	2.98	7.52	9.08	0.80	7.52	10.6	< .001
7Y	6	5.59	1.24	3.07	8.11	8.75	0.99	6.80	10.7	< .001
8Y	5	4.29	1.29	1.68	6.91	10.0	1.05	7.97	12.1	< .001
9Y	5	4.39	1.29	1.78	7.01	9.94	1.05	7.87	12.0	< .001
10Y	5	3.49	1.29	0.88	6.11	10.8	1.05	8.77	12.9	< .001

*SD* standard deviation, *CI* confidence intervals, ^*^P value Mixed-effects models

### Changes in IOP after initiation of tacrolimus eye drop treatment

**Fig. 4:**
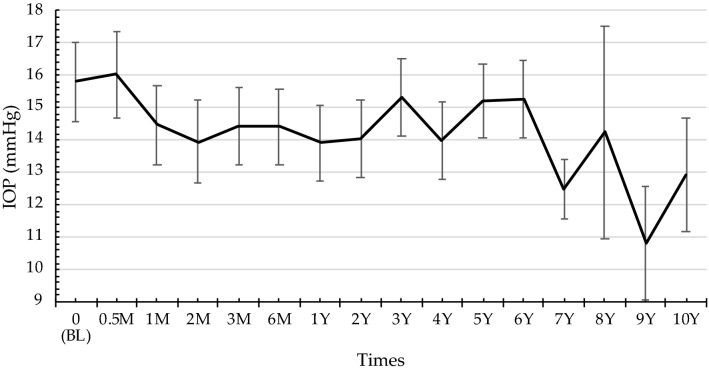
Changes in intraocular pressure after initiation of tacrolimus eye drop treatment. IOP gradually decreased from baseline to 2 weeks after beginning tacrolimus eye drop treatment. Tacrolimus eye drops elicited a statistically significant difference in the mean IOP over the course of treatment

Changes in IOP over the study period are shown in Fig. 4. IOP gradually decreased from 1 month after starting tacrolimus eye drop treatment. Tacrolimus eye drops elicited statistically significant differences in mean IOP over the course of treatment (P < 0.001).

### Changes in steroid use in combination with tacrolimus

The percentage of eyes using topical steroids and/or oral steroid decreased from baseline after starting tacrolimus treatment. Of 6 patients who required treatment with topical steroids, 50% (3 patients) were successfully weaned off topical steroids to tacrolimus treatment by 1 year (2 patients from 2 weeks, and 1 patient from 1 year). In the other 3 patients (50%), they were unable to wean off topical steroids completely over the time course of treatment.

### Adverse reactions

Adverse reactions are shown in Table 3 with reference to previous report [16]. All patients complained of a mild, well-tolerated burning sensation upon application of the eye drops. Although 1 other patient who was treated with tacrolimus eye drop alone had bacterial keratitis, no other patients had any infectious adverse events after initiation of tacrolimus eye drop use. When we observed 2 patients treated with both topical steroids and topical tacrolimus tended to develop increased IOP, we stopped steroid and carried out tacrolimus eye drop alone. Thereafter, IOP decreased and remained within the normal range.

**Table 3:** Adverse events

Incidences	Eyes (%)
Elevated IOP	4 (33.3)
Hordeolum	0 (0)
Chalazion	0 (0)
Lid herpes	0 (0)
Herpetic epithelial keratitis	0 (0)
Bacterial keratitis	1 (8.3)
Corneal erosion	0 (0)

## Discussion

In this long-term, retrospective study, we investigated changes in clinical signs and IOP due to using tacrolimus eye drops for more than 6 years. We found that topical tacrolimus had immediate and continuous beneficial effects on the clinical outcomes of these eyes. Although AKC and VKC mechanistically and partially differ from perennial allergic conjunctivitis (e.g., related to house dust and pollutants), seasonal allergic conjunctivitis (e.g., related to pollen), and giant papillary conjunctivitis (e.g., related to contact lens wearing), these can be cofactors in deterioration. [17, 18]. In our experience of treating patients with AKC and VKC, we have encountered cases that were extremely resistant to conventional treatments. Since 2005, 0.1% cyclosporine aqueous ophthalmic solution became available and there were some reports on its therapeutic effects on conventional treatment-resistant disease [21–23]. Cyclosporine binds to cyclophilin, while tacrolimus binds to FK506 protein; these agents exert pharmacological activities, such as inhibition of cytokines (e.g., interleukin [IL]-2, IL-4, IL-5, and interferon-γ) production and suppression of mast cell degranulation, by inhibition of calcineurin activation. Tacrolimus has also been reported to inhibit calcineurin approximately 100 times more effectively than cyclosporine [24]. Although both of these nonsteroidal immunomodulators can result in an intense stinging sensation, leading to poor compliance [16, 22, 25], all patients in this study tolerated this sensation well. Several reports have described the effect of tacrolimus ointment [25–30] and ophthalmic suspension [11, 31] on ACDs. On the other hand, VKC is a disease with repeated exacerbations and remissions from childhood through adolescence. In addition, AKC may require more than a few years of treatment because the underlying AD is not completely cured. Thus, this study is considered to be meaningful because it evaluates a longer course of treatment. However, we noted that medical records were not adequately kept, because mild VKC healed naturally with maturation, or because the patient relocated, and thus their visits to the hospital were suspended. It was also difficult to evaluate the data retrospectively in young, critically patients because eyelid opening was difficult, and the eyes could not be easily observed and photographed. Hence, we could only assess 12 cases of AKC and VKC.

In this study, 3 cases deteriorated over time in spite of the combination therapy. Because they were young, it was possible that their eye drop use compliance was poor, or that clinical deterioration occurred due to sporting activities and/or antigen exposure. In such cases, our previous report on eye washing may also be useful [32]. Additionally, in another patient (a 31-year-old male) with AKC, the ocular findings also worsened when his facial AD worsened. Epithelial and epidermal cytokines (e.g., IL-25, IL-33, and thymic stromal lymphopoietin [TSLP]) released in AD skin lesions can further stimulate group 2 innate lymphoid cells (ILC2) to release type 2 cytokines (e.g., IL-5 and IL-13) [33, 34]. Thus, it is necessary to corroborate not only the ocular findings, but also the dermatological findings when treating AKC.

As previously mentioned, the borderline of definitive diagnosis between AKC and VKC is vague in actual clinical practice. The Japanese guidelines define VKC patients as a mixture of those with and without AD. However, a large number of patients diagnosed with VKC with AD may be AKC. In addition, there is no regulation on the site of AD. Therefore, in the present study, all patients had AD, but AKC and VKC were mixed because AKC was defined as those with AD on the face. For example, it is strange that VKC diagnosed in young patients with atopic changes on their face may transition to AKC during maturation. Thus, we also evaluated the time course difference in the total scores and the IOP among these groups (Additional file 2: Figure S1 and Additional file 3: Figure S2). Because the mean use period of tacrolimus in the VKC group was 6.4 ± 0.7 years, the comparison between the two groups was up to 7 years. The period after 7 years reflects the results of only some cases of AKC, but both groups have significantly improved and remained stable over time. Although VKC often spontaneously remits in adolescence, it is valuable to suggest that it is stable for as long as 10 years in severe ACDs, including refractory AKC cases. However, this study was a retrospective study, and few clear image data remained because of long-term observation. These are very rare diseases, but we believe that if there are enough cases that can be compared between the AKC and VKC groups, future studies will ascertain a clearer distinction between AKC and VKC.

Steroids may elevate IOP, but IOP is not likely to elevate during treatment with tacrolimus, as previously reported [25]. In fact, we found that 2 cases had elevated IOP (Table 3); however, the concomitant use of steroids, the poor condition of the eyelid (e.g., lid swelling and hard skin), or secondary difficulty in lid opening or closure may have influenced this finding. Therefore, we considered that the elevated IOP in these cases was due to the influence of the eyelids and steroid use after initiation of tacrolimus. In other 10 cases, reduction or discontinuation of steroids or improvement of eyelid findings may have resulted in reduction or stabilization of IOP. Clinicians tend to use topical steroids first and add tacrolimus eye drops as an adjunctive treatment. In fact, because pretreatment with steroids before the initiation of topical tacrolimus or combination therapy after the initiation of topical tacrolimus improved clinical signs, the possibility of a synergistic effect of tacrolimus with steroids cannot be ruled out. Moreover, as is known, there are individual differences in the ease with which steroids increase IOP [35]. One of these 10 patients received topical steroids twice a day until his last visit, but IOP was not elevated.

The current study had several limitations. First, the number of patients in this study was small. There were few cases with clear medical records and images. We found only one case of ocular infection. Because the risk of infection and elevated IOP should be considered more carefully in cases with combination therapy involving steroids, it is necessary to examine which condition is prone to infection after the initiation of tacrolimus treatment in the future study. Following 12 patients even for on average 8 years would only rule out side effects that have a frequency of at least 25%. Therefore, side effects that occur less than 25% of the time could be missed. However, our results provide the evidence supporting extremely long-term treatment of eyes with severe AKC and VKC with topical tacrolimus; such data have not been reported previously. Second, the initiation of topical tacrolimus differed among patients. As is known, symptoms are more sensitive to seasonal pollen and humidity. Ethically, treatment begins when patients are confronted with a worsening condition. In addition, because this study was retrospective and AKC and VKC were rare diseases, it was difficult to align the baseline period. Therefore, it is ideal to perform baseline measurements at the same time and to conduct a prospective study near future. However, In Japan, approval of specified clinical research started since 2018 is required to conduct this study as a prospective study. Thus, it cannot be conducted at Tsurumi University and Ryogoku Eye Clinic, which are not accredited institutions. But in this study, data were collected from two centers most using tacrolimus eye drops in Japan. Hence, we believe this is an invaluable long-term result even in a retrospective study. Third, we did not evaluate subjective symptoms (i.e., itching, pain, discharge, lacrimation, photophobia, and foreign body sensation). The correlation between the severity of subjective and objective clinical signs should be investigated in future.

## Conclusions

The results of this study suggest that 0.1% tacrolimus eye drops may effectively yield long-term improvement of clinical signs of severe VKC and AKC cases that are refractory to conventional treatments. When the condition improves, the number of eye drops used can be gradually decreased. Moreover, proactive tacrolimus therapy may also be effective for treatment of severe ACDs.

## Supplementary Information

**Additional file 1: Table S1.** Grading scores of ten clinical signs.

**Additional file 2: Figure S1.** Comparison of the scores of objective clinical signs in the AKC and VKC groups*. *Significantly higher scores were observed after initiating tacrolimus eye drops in AKC group than VKC group at 2 weeks and 2months (P=0 .02 and P=0.002, respectively).

**Additional file 3: Figure S2.** Comparison of intraocular pressure in the AKC and VKC groups*.* There were no significant differences among these groups.

## Data Availability

Data and materials are available upon request from the corresponding author at g.h.yazu@gmail.com.

## Abbreviations

ACDs: Allergic conjunctival diseases
AD: Atopic dermatitis
AKC: Atopic keratoconjunctivitis
BRM: Biologic response modifiers
CI: Confidence intervals
GPC: Giant papillary conjunctivitis
IFN: Interferon
IL: Interleukin
IMT: Immunomodulatory therapy
IOP: Intraocular pressure
NSAIDs: Non-steroidal anti-inflammatory drugs
PAC: Perennial allergic conjunctivitis
SAC: Seasonal allergic conjunctivitis;
SD: Standard deviation
VKC: Vernal keratoconjunctivitis
