# Supplementary material for: Long-term outcomes of 0.1% tacrolimus eye drops in eyes with severe allergic conjunctival diseases
Source: Allergy Asthma Clin Immunol. 2021 Feb 1;17:11. doi: 10.1186/s13223-021-00513-w (PMC7852099; doi:10.1186/s13223-021-00513-w)
Supplement: Supplementary file 1 — Additional file 1: Table S1. Grading scores of ten clinical signs. [file 13223_2021_513_MOESM1_ESM.docx]

Supplementary Table 1. Grading scores of ten clinical signs


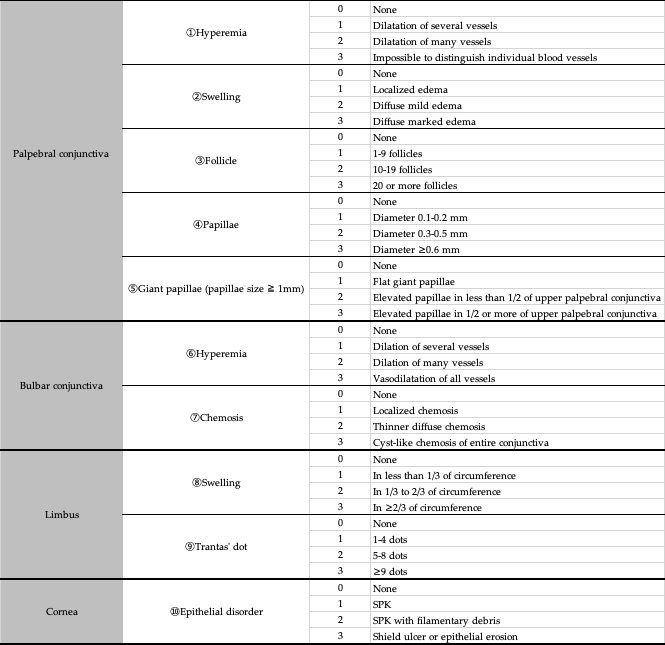


SPK: Superficial punctate keratitis
