# Supplementary material for: Long-term outcomes of 0.1% tacrolimus eye drops in eyes with severe allergic conjunctival diseases
Source: Allergy Asthma Clin Immunol. 2021 Feb 1;17:11. doi: 10.1186/s13223-021-00513-w (PMC7852099; doi:10.1186/s13223-021-00513-w)
Supplement: Supplementary file 2 [file 13223_2021_513_MOESM2_ESM.docx]

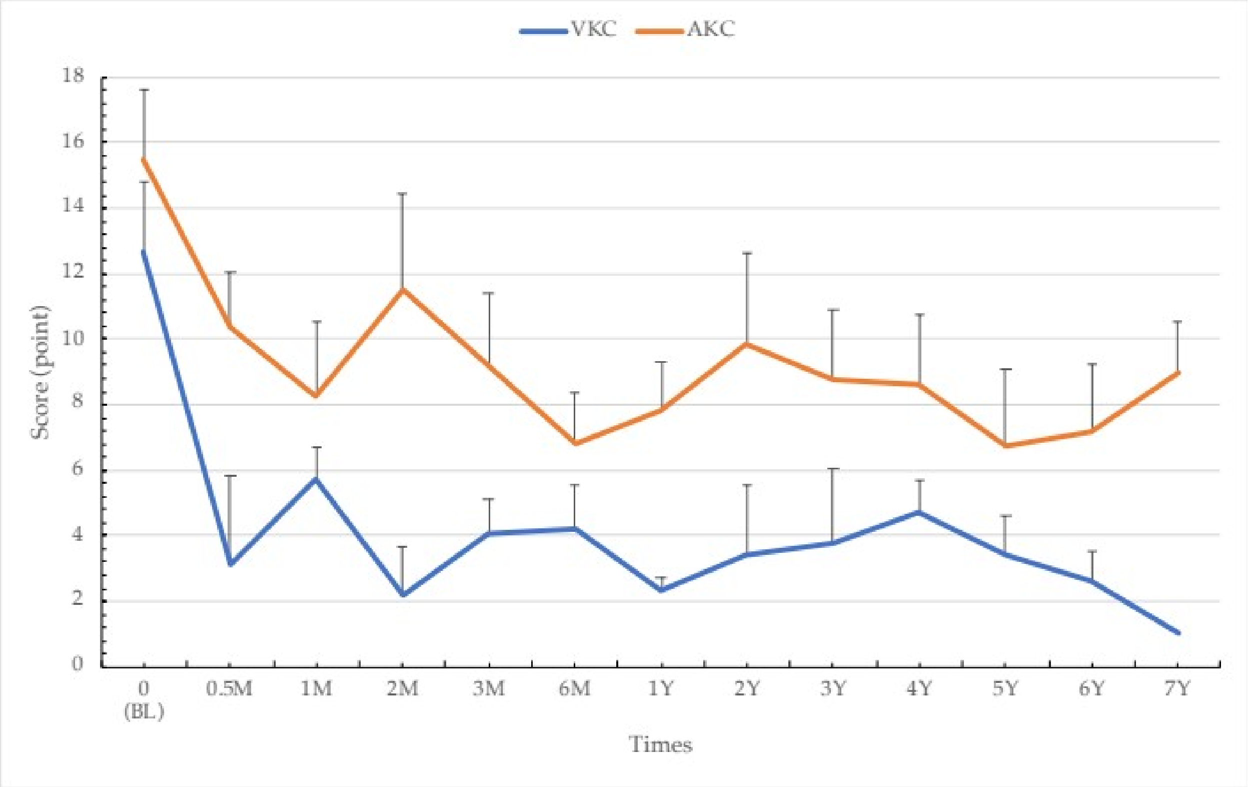

VKC
AKC
Score (point)
18
16
14
12
10
8
6
4
2
0
0
(BL)
0.5M
1M
2M
3M
6M
1Y
2Y
3Y
4Y
5Y
6Y
7Y
Times
